# Introducing rapid oral–fluid HIV testing among high risk populations in Shandong, China: feasibility and challenges

**DOI:** 10.1186/1471-2458-14-422

**Published:** 2014-05-03

**Authors:** Gifty Marley, Dianmin Kang, Erin C Wilson, Tao Huang, Yuesheng Qian, Xiufang Li, Xiaorun Tao, Guoyong Wang, Huanmiao Xun, Wei Ma

**Affiliations:** 1 Department of Epidemiology and Health Statistics, School of Public Health, Shandong University, #44 West Wenhua Road, Jinan 250012, Shandong Province, PR China; 2 Shandong Provincial Center for Disease Control and Prevention, Jinan, China; 3 San Francisco Department of Public Health, San Francisco, CA, USA; 4 The Sex Health Center, The Affiliated Hospital of Medical College Qingdao University, Qingdao, China

**Keywords:** HIV/AIDS, Rapid oral fluid test, Saliva testing, Feasibility and acceptance, China

## Abstract

**Background:**

This study was conducted to ascertain the feasibility of using rapid oral fluid testing as an alternative HIV testing method in China.

**Method:**

This is a mixed-method study among men who have sex with men (MSM), female sex workers (FSW) and VCT clients, conducted in 4 cities in Shandong Province. A pre-tested questionnaire was administered to 1137 participants through face-to-face interview to assess demographic characteristics, HIV testing histories and willingness to accept rapid oral fluid testing. VCT clients were provided with the saliva test kits for a screening test and errors in operation were recorded. Testing results were compared between oral and blood testing. Short feedback questionnaire was administered to 200 FSW who had undergone oral testing.

**Results:**

The rate of willingness to take oral-fluid HIV testing among MSM, FSW and VCT clients was 72.8%, 72.1% and 67.4% respectively. Common errors recorded during test kit operation by the 229 VCT clients included: unpreparedness, wrong swab sampling, wrong dilution, wrong testing and inability to read test results. Advantages of oral testing listed by participants included: less intrusive, painlessness, easy self- testing and privacy. Disadvantages included perceived unreliable results (55.5%) and not nationally recognised (9%). Comparison of saliva and the blood testing results recorded a consistency rate of 0.970 (*χ*^2^ = 153.348, *P <* 0.001), implying an excellent consistency.

**Conclusion:**

Introduction of oral rapid fluid testing as an alternative HIV testing method in China is highly feasible but with some challenges including low recognition and operation errors.

## Background

It is estimated that by the end of 2011, the number of people living with HIV in China was about 780,000 [1]. The National HIV/AIDs report showed that, men who have sex with men (MSM) and female sex workers FSW recorded a 5% and 6% (median) estimated prevalence respectively in 2009 which far exceeds the general populations’ prevalence of 0.05% [2,3]. Hence, MSM and FSW are categorized as ‘populations at highest risk’ of infection for HIV in China. Report at the end of October 2012 showed that cumulatively, the number of reported HIV/AIDS cases was 492, 191, with 383,285 people living with HIV/AIDS [4]. This means that over half of people living with HIV/AIDS do not know that they are infected. Studies showed that about 33.2% of MSM and 42% of FSW have never been tested [3,5] and 86.1% of infected MSM are unaware of their infection status [5-7]. Thus, testing rate among both MSM and FSW is low and this means that over half of people living with HIV/AIDS do not know that they are infected [5-9].

Voluntary counseling and testing (VCT) has been an important tool employed by WHO and other health organizations worldwide to identify new cases and implement treatment as prevention strategies [10]. The imperative of scaling up HIV testing in China was recognized as an important strategy for identifying unknown positives and preventing onward transmission of HIV [11,12]. Free HIV testing has been made available, and expanded from 365 counties in 15 provinces in 2002 to over 2300 counties, with 3037 sites, in all provinces by 2006 [1,4,11]. However, the biological, social, economic, political, and ethical concerns surrounding HIV surveillance, access to care and prevention still remains complex [1,2,13].

Currently, enzyme-linked immunosorbent assay (ELISA) combined with western-blot and rapid blood testing are the major HIV antibody testing methods employed in HIV testing in most countries including China [14-16]. Though ELISA is the ‘gold-standard’ HIV testing method for screening, which is recognized traditionally, it is not without disadvantages [17-19]. For example, it cannot be used in point-of care since it requires ELISA equipment and professional staff for its use, waiting time for results has been reported to be too long and expensive hence discouraging users [20]. Also, the use of needles and sharp objects with their associated pains to obtain the needed blood sample deter many people from using it. For FSW and MSM, providing an alternative testing method eliminates the issue of facing professionals in front of whom they might have to answer questions on their number of sexual partners and their sexual orientation which most of them find uncomfortable [2,3,5-9]. Notwithstanding there is an urgent need for acceptable and feasible testing methods among MSM, FSW and VCT clients in order to scale up HIV testing.

Rapid oral fluid based testing method has already been approved and is in use in most HIV testing institutes worldwide [7,21-25]. The testing kits and testing method have further been found to be accurate, much safer and easier to use and therefore recommended for adoption and use in the developing countries due to its simplicity, versatility and, feasibility that enables easier implementation and use even in rural areas and private health institutions [22,23]. Also, incorporation of oral HIV tests could form part of a multi-pronged prevention strategy in transforming the trajectory of the HIV epidemic in China and possibly the world at large [24,25]. A study conducted in Seattle indicated that clients are more likely to accept HIV testing using this method instead of standard blood testing because it is less intrusive and painless [26].

Although four testing kits have been approved as of the end of 2011; [27] “ Sidaizhiyu Oral Fluid HIV ½ Antibody Diagnostic Kit” (Wondfo Biomedical Co., Ltd, 2011), “Union Oral Mucosal Transudate HIV-1/2 Antibody Detection Kit “(Chengdu Union Bio-technology Ltd., 2011), “Aware HIV-1/2 OMT” (Beijing Marrbio Ltd, 2008) and “HIV 1/2 antibody Dot ELISA rapid test” (Beijing Wantai Biological Pharmacy Enterprise Co., Ltd., 2011 ), [28] rapid oral testing is however not been widely used and no data exist to assess the uptake of oral testing and how accurate these kits are in real world settings in China. Hence this studies to ascertain the feasibility of up scaling its usage in China.

## Methods

### Design

From July 2011 to December 2012, we conducted a mixed-method study among 3 major high risk populations in 4 cities in Shandong Province, China, based on the Centre for Disease Control (CDC) implementation units for the province and the location of VCT centres.

### Setting

Men who have sex with men (MSM), female sex workers (FSW) and VCT clients were recruited from Qingdao City, Yantai City, Zibo City and Jiaozhou County of Qingdao City. FSW participants were recruited from their workplace, including massage centers, salons (FSWs), bars (some MSM were found here), hotels, restaurants, and construction sites. Some MSM were recruited in workshops convened for AIDS education or the office of a local nongovernmental organization run by MSM. Potential participants were approached either through managers of their workplace or through organizers of workshops. VCT clients were recruited from VCT clinics in Qingdao City and Yantai City.

### Participants

Inclusion criteria for the study were as follows: 1) 18 years of age and older; 2) willingness to participate in the survey; 3) had sex (either anal or oral or both) with a man (men) in the past 12 months (for MSM); 4) provided commercial sex (traded sex for money or goods) in the past 12 months (for FSW); 5) visited VCT clinics for HIV counselling and/or testing (for VCT clients). Anonymous feedback questionnaire with six opened questions to be answered participants were administered to assess possible feasibility of introducing the oral fluid test.

### Assessment of attitudes and feasibility of rapid oral tests

Three sources of data were gathered from all participants to assess attitudes and feasibility of rapid oral tests in the real world settings in China: (1) data from a pre oral rapid HIV test survey to assess feasibility and acceptability of oral rapid HIV tests among MSM, FSW and VCT clients; (2) observations among VCT clients to assess the quality of self- delivery of the oral rapid test by participants and professional re-testing to assess test accuracy, and (3) data from an open-ended survey with FSW to assess their attitudes towards the test after getting oral HIV testing.

First, a pre oral rapid HIV test questionnaire was conducted to assess acceptance willingness of rapid oral testing within these three populations. The pre-tested questionnaire administered to all participants assessed demographic characteristics, attitudes towards HIV testing, willingness to accept rapid oral testing, and feasibility. To assess feasibility and acceptability, we assessed participant’s history of HIV antibody testing, previous testing methods known to the participants, knowledge about HIV saliva testing before this survey and what/who will influence their decision to ever opt for HIV saliva testing. Acceptability was assessed through 5 questioning items: (1) knowledge about HIV saliva testing before this survey; (2) if previously tested with saliva test kit; (3) willingness to accept saliva rapid test in future; (4) willingness to accept saliva rapid test even if the accuracy of the saliva test is lower compared to blood test; and (5) reasons for willingness to accept saliva testing. All information was obtained through direct face-to-face interviews. Subjects were consented by obtaining their written informed consent for voluntary rapid oral fluid testing and participation in the pre oral test questionnaire. The questionnaire was designed and administered to ensure information privacy and subject’s comfort with the questions involved.

A total of 229 VCT clients who agreed to partake in the study after going through counseling were provided with the saliva test kit to use for the screening test and their results recorded. The VCT clients operated the testing by themselves after reading the instruction provided by test kits. Their ability to use the new test kit effectively and correctly was keenly observed by research staff during the testing process and the errors they committed while operating the kits were recorded. A consultant was then made to re-test the participants using the blood testing method and the results recorded. The data from the oral and blood tests among 340 VCT clients was then statistically analyzed to determine consistency between results.

Finally, 200 FSW underwent oral rapid testing by health staff and were interviewed by research staff immediately after being tested to gather data on acceptability of the oral HIV testing and home testing for HIV. Also, this assessment was conducted to measure the possibility of enhancing home-based and self-testing. Questions asked included thoughts on HIV saliva testing benefits compared to blood-based testing; testing method preference after experiencing saliva testing; their willingness to accept home based/self-testing and suggestions or recommendations to enhance acceptability of saliva testing.

### Ethical approval

This study was approved by the ethics committee of School of Public Health, Shandong University before it was conducted. Ethical approval identification number was 201110302.

### Statistical analysis

All data were double entered with EpiData 3.1 software (Denmark) and discrepancies were checked against the raw data. Data analysis was performed using the SAS 9.1. For univariant analysis, *χ*^2^ and Fisher’s exact test was performed and a *P* value less than 0.05 was considered statistically significant. Also, sensitivity and specificity of the test kit was calculated to control the rate of misdiagnosis in addition to a validity and accuracy test conducted to authenticate all results. The McNemar test was used to compare test results obtained from the participants and that from the consultant; this was to measure consistency between the rapid oral fluid testing results and the blood-based testing results. The kappa statistic was also calculated for the observed consistency and the valued was recorded.

## Results

There were 371 MSM, 405 FSW and 361 VCT clients with participation rates of 98.9%, 99.3% and 98.1% respectively.

### Acceptance among all three populations

The collected information showed that, more than half of the study population are willing to accept and opt for oral rapid testing at their preferred testing venues. By population, the acceptance willingness rate among MSM, FSW and VCT clients was 72.8%, 72.1% and 67.4% respectively [25]. Chi-square (*χ*^*2*^) on the observed rates recorded a *P*-value of 0.133 indicating no statistical significance in acceptance levels between the 3 high risk groups. Among those willing to accept oral rapid fluid testing, MSM reported the highest willingness rate (77.2%), compared to VCT clients and FSW (72.1% and 61.4% respectively) irrespective of the possible limitations of the testing kit (*P* <0.001).

### Kit operation among VCT clients

Common errors in kit operation observed were: unpreparedness before start, inability to take swab sample correctly, dilution, testing and reading of the test results. Out of the 229 VCT clients that underwent testing, only 133 (58%) were correctly prepared by reading the instruction manual before the next step. Twenty-three (10.1%) of the participants were unable to take correct swab samples as per the instruction manual (subjects directly wiping sampling swab on the teeth back and forth error recorded the highest frequency of 19). During dilution, the most frequent error was subjects not scratching and squeezing the oral swab properly (34 subjects). Only 4 participants failed to locate or take out the test paper and 1 person touched the testing area with fingers during testing. In the end, 40 of the total participants could not read or interpret their results without the help of a consultant, 9 could not do the results reading since they did not read the manual in preparation and 8 participants did not want to know their result saying they were too scared (Table 1). Consistency analysis (using the McNemar-test to compare the subjects’ self test results to the consultants’ test results) recorded a kappa value of 0.551 (*χ*^*2*^ = 11.000 ~ *P* = 0.012). This implies that most participants were able to correctly read their results equally as the medical consultant did (Table 2).

**Table 1:** Major errors committed by 229 VCT clients during the saliva testing screening process in Shandong Province, China

**Steps**	**Wrongly done**	**Percent (%)**	**Errors**	
			**Main problems**	**Frequency**
**Preparation**	94	41.4	No preparation	62
			Opened the oral swab directly	16
			Didn’t open the buffer tube	13
			Damping fluid was not well shaken	10
**Taking sample**	23	10.1	Wiped sampling swab on the teeth back and forth	19
			Put into the mouth directly to take sampling or spit saliva directly into the buffer tube	3
**Sample dilution**	36	15.9	Didn’t’t scratch and squeeze oral swab	34
			Tilted buffer tube	1
**Testing process**	9	4	Couldn’t find or take out the test paper	4
			Touched testing area of test paper	1
**Reading results**	8	3.5	Needed guidance of consultants	40
			Didn’t’t read the introduction book	9
			Too scared to see the results	8

**Table 2:** Consistency for observed saliva testing results: VCT clients vs. Consultant (McNemar-test)

		**Consultant**			**Total**
		**1**	**2**	**3**	
**Clients**	**1**	6	3	0	9
	**2**	0	209	3	212
	**3**	7	1	0	8
**Total**		13	213	3	229

Kappa = 0.551, *χ*^*2*^ = 11.000, *P* =0.012.

Key: 1 = positive 2 = negative 3 = indeterminate.

### Validity

Analysis comparing results from the saliva test kit versus results from the blood test recorded a consistency rate of 0.970 (*χ*^*2*^ = 153.348 ~ *P <* 0.001). The saliva test kit showed a sensitivity of 77.5%, a specificity of 99.76% and a 0.33% rate of misdiagnosis, with a positive predictive value (PPV) and a negative predictive value (NPV) of 0.969 and 0.971 respectively. The two tests generally recorded a very high accuracy value of 0.980 (Table 3).

**Table 3:** Validity and accuracy analysis of the Saliva test (screening process) results versus the blood test (“golden standard”) results collected from the 340 VCT clients

		**Blood testing**		**Total**
		**Positive**	**Negative**	
Saliva testing	Positive	31	1	32
	Negative	9	299	308
Total		40	300	340

Sensitivity (true positive rate) = 0.775.

Specificity (true negative rate) = 0.997.

Rate of misdiagnosis (false positive rate) = 0.003.

Positive predictive value (PPV) = 0.969.

Negative predictive value (NPV) = 0.971.

Accuracy (ACC) = 0.980.

Consistency rate = 0.970.

### Feedback after testing among FSW

Open ended responses from the 200 FSW regarding feasibility were the following; the primary advantages FSW saw of the oral rapid test were that no blood is involved in the testing 193 (96.5%), the test is pain free 191 (95.5%), the test is recommended by medical professionals 68 (34%) and would use it if it was routinely offered in HIV testing facilities 28 (14%). For disadvantages, 82 (41%) participants found no disadvantages, 111 (55.5%) doubted the accuracy of results, and 18 (9%) did not think the tests were nationally recognised, thus, they have not heard of any laws or advertisement by their ministry of health suggesting that the testing method is been approved/endorsed as an official testing method for use in testing centres. Despite the disadvantages, 83 (42.8%) chose saliva testing when asked their preference for an HIV testing method, while 111 (57.2%) still opted for blood testing as their preference. FSW gave a number of recommendations for increasing feasibility and acceptability of the oral rapid HIV test which included increased results accuracy (n = 66, 33%), 15 (7.5%) wanted the test kit operating steps simplified and 14 (7%) wanted the test to be offered at no cost to the VCT clients. Finally, 108 (54.5%) opted for self-test at home when given other testing venue site choices (Table 4).

**Table 4:** Feedback and recommendations made by 200 of the total FSW who underwent the saliva testing

	**Frequency**	**Percentage (%)**
**Advantages**		
National recognition	4	2
Common use	28	14
Accurate and reliable	26	13
No blood	193	96.5
No pain	191	95.5
Short waiting time for results	5	2.5
All friends use	1	0.5
Friends to recommend	16	8
Consultant recommend	68	34
It does not matter	1	0.5
**Disadvantages**		
None	82	41
Complicated kit operation	3	1.5
Results not credible	111	55.5
Results not recognised	18	9
Expensive	1	0.5
Testing method preference		
Saliva testing	83	42.8
Blood testing	111	57.2
**Need improvement**		
None	108	54
Simplify kit operation	14	7
Enhance results accuracy	66	33
Cost free	15	7.5
**HIV home self testing preference**		
Yes	108	54.5
No	90	45.5

## Discussion

A key finding that may impact the scale up of rapid oral HIV testing was that the test kit recorded lower sensitivity (77.5%) than stated by the manufactures, which may reduce credibility of this test and consequently, acceptability by populations at risk for HIV. A number of factors that may explain our observed low sensitivity include the small sample size and the many errors made by participants during the testing phase rendering most of the test results invalid. This could have also decreased the sensitivity of the test kit and thereby slightly lowering the positive predictive value (PPV) from the manufacturers stated value of 99% PPV. The comparison between consultant testing and client self-test recorded a low sensitivity of for self-testers. This observation could be due to errors committed by the clients during the testing process leading to the indeterminate results, invalid tests and missing data. The issue of indeterminate results and invalid tests which lowers the sensitivity of the test is a real life problem. Hence client with such results are encouraged to retest and also seek assistance from consultants. Also, the sensitivity of self-test may vary cross population, depending on the populations’ educational level and their ability to learn quickly [27].

Similar studies on the use of HIV rapid oral fluid test for self-testing have been conducted in the U.S. and some parts of Africa [17-24]. Most of those studies concluded from their obtained results that, the idea of using saliva testing as an alternative testing method nationally was highly feasible if only the populations’ knowledge of how to use the test kit and its limitations is raised through education before implementation [19-26]. The US studies was conducted using a rapid saliva test kit known as “OraQuick test (OraSure technologies)” that recorded a sensitivity of 97.4% and specificity of 99% [26,29], though their observed sensitivity of 97.4% was somewhat greater than our observed sensitivity of 77.5%, the general conclusions we both derived from the studies are similar. Additionally, the use of rapid oral fluid testing as a testing or screening method for HIV antibodies was found to be safe and highly effective, thus highly feasible in a number of countries [19-25,30]. Findings from the descriptive analysis of our study suggests that, individuals from populations at risk for HIV determine what HIV test they will take based on accurate results, needs for privacy, and the cost of testing. To address the concern about accuracy, a validity and consistency analysis was conducted on the test results. Our analysis recorded a consistency rate of 0.970 with a kappa value of 0.551, indicating an excellent consistency. This consistency rate implies that the test results from the saliva test kit were both consistent with the results from the blood test and equally credible and accurate as other testing methods like blood tests.

Further analysis to assess feasibility depended on participants’ ability to operate the test kit and read results correctly. Observations made during the testing process showed that about half of all participants found the directions to be too complicated, which accounted for most of the errors observed during the process. For example, 40 (17.47%) did not want to watch or read the introduction book and reported that it was too complicated and with specialized language that they did not understand. These individuals had to go through the testing process with the assistance of medical professionals, 1(0.45%) touched the test area of test paper with the hand while running the test, 1(0.45%) tore off the middle membrane of test paper, and 4 (1.82%) could not find or take out the test paper. All these errors impacted the sensitivity and accuracy rate of the test kit itself. These errors could be eliminated through educational workshops and media education on the credibility and accuracy of the test kit, and through the development of simpler operational steps on how to operate the test kit and interpret results. Videos that walk testers through the steps in testing could be useful as well.

When questioned on testing venue preferences, 519 (52.7%) subjects wanted to be tested at the CDC office that currently provides HIV testing services to the public and 277 (24.5%) preferred to be tested in the hospital. HIV home testing was preferred by 353 (57.3%) participants because it was convenient, and 360 (58.4%) participants wanted home testing for privacy reasons. Another study among Chinese MSM also found that MSM prefer to test at a VCT clinic opened by a local CDC rather than a hospital [6]. Thus, many participants were willing to accept the use of the rapid oral fluid HIV testing, which suggests high feasibility in the population. However, we did find that the thought of results not being credible and testing method not being nationally recognised as participants put it explains why many participants are hesitant to pick this method as their HIV testing method of choice.

This study has a few limitations. Some of the subjects refused to answer questions required for the descriptive analysis leading to missing data which may or may not have affected the overall study results. This was attributed to reasons like them not knowing the answer to the question or did not really understand the question. Also, could be that they were uncomfortable with how personal the question was. A total of 6 missing data points representing 3% was recorded. Another limitation was limited budget constraint which affected the total number of people we could sample and test.

## Conclusion

The findings from this study indicate that the introduction of rapid oral fluid HIV testing (saliva testing) as an additional testing or screening method in these cities is highly feasible. However, challenges still exist, for example, the level of knowledge on rapid oral fluid testing is low among the high risk population groups; operational errors influence the accuracy and sensitivity of the test. Hence, there is a need for mass education before the implementation of this new method. One option for mass education may be an easy-to-understand video that can be shared online to increase awareness and accuracy. Finally, additional research should be conducted to understand feasibility in other parts of the country.

## Competing interests

The authors declare that they have no competing interests.

## Authors’ contributions

WM, DK, YQ, XT and GW conceived and designed the experiments. XT, TH, XL, YQ and GW performed the experiments. GM, HX and EW analyzed the data. GM, HX and WM wrote the paper. GM, EW, DK and WM critically revised the paper. All authors read and approved the final manuscript.

## Pre-publication history

The pre-publication history for this paper can be accessed here:

http://www.biomedcentral.com/1471-2458/14/422/prepub
